# Rtf1 Transcriptionally Regulates Neonatal and Adult Cardiomyocyte Biology

**DOI:** 10.3390/jcdd10050221

**Published:** 2023-05-20

**Authors:** Adam D. Langenbacher, Fei Lu, Lauren Crisman, Zi Yi Stephanie Huang, Douglas J. Chapski, Thomas M. Vondriska, Yibin Wang, Chen Gao, Jau-Nian Chen

**Affiliations:** 1 Department of Molecular, Cell and Developmental Biology, University of California, Los Angeles, CA 90025, USAlcrisman30@g.ucla.edu (L.C.);; 2 Departments of Anesthesiology, Medicine, and Physiology, David Geffen School of Medicine, University of California, Los Angeles, CA 90025, USAtvondriska@mednet.ucla.edu (T.M.V.);; 3 Signature Research Program in Cardiovascular and Metabolic Diseases, Duke-NUS School of Medicine and National Heart Center of Singapore, Singapore 169857, Singapore; 4 Department of Pharmacology and Systems Physiology, University of Cincinnati College of Medicine, Cincinnati, OH 45267, USA

**Keywords:** cardiomyocyte, heart, Rtf1, PAF1 complex, transcription regulation

## Abstract

The PAF1 complex component Rtf1 is an RNA Polymerase II-interacting transcription regulatory protein that promotes transcription elongation and the co-transcriptional monoubiquitination of histone 2B. Rtf1 plays an essential role in the specification of cardiac progenitors from the lateral plate mesoderm during early embryogenesis, but its requirement in mature cardiac cells is unknown. Here, we investigate the importance of Rtf1 in neonatal and adult cardiomyocytes using knockdown and knockout approaches. We demonstrate that loss of Rtf1 activity in neonatal cardiomyocytes disrupts cell morphology and results in a breakdown of sarcomeres. Similarly, Rtf1 ablation in mature cardiomyocytes of the adult mouse heart leads to myofibril disorganization, disrupted cell–cell junctions, fibrosis, and systolic dysfunction. Rtf1 knockout hearts eventually fail and exhibit structural and gene expression defects resembling dilated cardiomyopathy. Intriguingly, we observed that loss of Rtf1 activity causes a rapid change in the expression of key cardiac structural and functional genes in both neonatal and adult cardiomyocytes, suggesting that Rtf1 is continuously required to support expression of the cardiac gene program.

## 1. Introduction

Transcription regulation involves the integration of a vast array of inputs, including activators and repressors, chromatin remodelers, histone modification readers and writers, pausing, elongation and termination factors, and other supporting complexes [1,2,3,4]. The Polymerase-Associated Factor 1 complex (PAF1C) is an RNA Polymerase II (RNA Pol II)-associated complex that regulates multiple steps in the transcription cycle via its direct interaction with the transcription apparatus and by serving as a platform for the binding of other regulatory complexes and transcription factors [5,6,7]. Five subunits, Paf1, Ctr9, Leo1, Cdc73, and Rtf1, constitute the evolutionarily conserved core of the PAF1C, while one additional protein, Ski8, associates with the human PAF1C [8,9,10,11]. Members of the PAF1C and their interacting partners have been implicated in biological processes ranging from embryonic development and stem cell biology to tumorigenesis [5,12,13,14,15,16,17,18,19,20,21,22,23,24,25,26]. The full extent of the PAF1C’s transcription regulatory activities and its impacts on development and disease remain an open question.

The PAF1C component Rtf1 is a transcription elongation factor that influences the translocation of RNA Pol II via several highly conserved functional domains. Structural and biochemical examination of the activated transcription complex containing RNA Pol II, DSIF, PAF1C, and Spt6 has revealed that Rtf1’s latch domain extends into a groove in RNA Pol II near the bridge helix and promotes the rate of elongation, suggesting that Rtf1 may directly stimulate translocation by facilitating bridge helix movements [27]. Additionally, through its Plus3 domain, Rtf1 directly interacts with the phosphorylated C-terminal domain of Spt5, a component of the DSIF complex and an established pro-elongation factor [28], but how this interaction might modulate transcription elongation at a mechanistic level is not yet known. Interestingly, Rtf1 can also promote RNA Pol II elongation velocity independent of the presence of other PAF1C components [29,30].

Rtf1 also facilitates co-transcriptional histone modification via a small domain known as the Histone Modification Domain (HMD). The HMD serves as an interaction site for the E2 ubiquitin conjugating enzyme Rad6 and also comes into contact with the nucleosome acidic patch in the cleft between histones 2B and 2A, potentially serving as a bridge between these proteins [31,32]. In yeast, complete loss of Rtf1 activity or mutation of the HMD leads to a reduction in histone H2B monoubiquitination (H2Bub1), along with decreases in histone marks supporting active transcription including trimethylated histone H3 lysine 4 (H3K4me3) and trimethylated histone H3 lysine 79 (H3K79me3) [15,33,34,35,36,37,38]. Similarly, overexpression of an Rtf1 deletion mutant lacking the HMD is unable to rescue H2B monoubiquination in human cells treated with Rtf1 shRNA [30]. On the other hand, overexpression of the 89 amino acid HMD in an Rtf1-deficient background can rescue H2B monoubiquitination [35], suggesting a functionally modular nature of the Rtf1 protein.

Consistent with Rtf1’s range of transcription regulatory roles, Rtf1 activity influences genes and pathways involved in the embryonic development of multiple tissues. Rtf1 activity negatively regulates erythroid gene expression and the development of the erythroid lineage [21], but positively regulates Notch signaling during zebrafish somitogenesis and *Drosophila* wing morphogenesis [14,15]. Rtf1 also antagonizes the activity of Cdk9/PTEF-b, thereby promoting pre-migratory neural crest maintenance [18]. Our lab has shown that during embryonic development, Rtf1 is essential for cardiogenesis. Embryos lacking Rtf1 form lateral plate mesoderm (LPM), the source of the cardiac lineage, but fail to produce any cardiac progenitor cells, resulting in a heartless embryo [19]. While these findings suggest that Rtf1 critically regulates cardiac identity during embryogenesis, the role of Rtf1 in the mature heart is unknown.

In this current study, we sought to determine the function of Rtf1 in neonatal and mature cardiac cells. We found that knockdown of Rtf1 in neonatal rat ventricular myocytes (NRVMs) disrupts their morphology, inducing abnormal cellular protrusions and a breakdown of sarcomeres. Similarly, knockout of Rtf1 in the adult mouse myocardium severely compromised cardiac structure and function, resulting in myofibril disorganization, disrupted cell–cell junctions, and fibrosis. Rtf1-deficient hearts fail within three weeks and exhibit a dilated left ventricle and systolic dysfunction. Intriguingly, we found that prior to the onset of functional deterioration, Rtf1-deficient hearts exhibit dysregulation of genes critically involved in calcium ion transport and the contractile machinery of cardiomyocytes. NRVMs likewise displayed changes in the expression of key cardiac structural and functional genes. Together, our data point to a crucial role for Rtf1 in maintaining the cardiac gene program in mature cardiomyocytes.

## 2. Materials and Methods

### 2.1. NRVM Culture

Neonatal rat ventricular myocytes (NRVMs) were isolated from post-natal P1-P3 day old Sprague–Dawley rat pups of mixed gender by UCLA Cardiovascular Research Theme Core services. NRVMs were cultured in DMEM-F12 medium (ThermoFisher #11330032, Waltham, MA, USA) with 10% horse serum (Sigma #H1138, St. Louis, MO, USA) and 1× Penicillin-Streptomycin (ThermoFisher #15140122) on plates precoated with 0.3% gelatin. Cells were changed into antibiotic-free and serum-free media (with 1% ITS) prior to transfection with siRNAs using Lipofectamine RNAiMAX (ThermoFisher #13778150). A total of 1 million cells were plated per well of a 6-well plate and were transfected with 40 pmol of siRNA. siRNAs used included MISSION siRNA Universal Negative Control #1 (Sigma #SIC001) and Rtf1 siRNA (Millipore-Sigma #SASI_Mm02_00336498, Burlington, MA, USA).

### 2.2. NRVM Immunostaining

NRVMs were fixed in 4% paraformaldehyde and permeabilized using acetone. Cells were incubated with α-actinin antibody (1:1000, clone EA-53, #A7732, Sigma-Aldrich, St. Louis, MO, USA) overnight at 4 °C. After washing with PBS, sections were incubated with fluorescently labeled secondary antibody and Phalloidin (ThermoFisher, #B3475) for 2 h. Cells were mounted using VectaShield fluorescence mounting medium with DAPI (Vector Laboratories) and imaged on a Zeiss LSM 800 confocal microscope.

### 2.3. RNA Isolation

RNA was isolated from mouse heart left ventricle tissue and NRVM cells with Trizol (ThermoFisher) and cleaned up using the Nucleospin RNA kit with on-column DNase digestion (Machery Nagel).

### 2.4. Real-Time Quantitative PCR

cDNA was synthesized with the iScript cDNA Synthesis Kit from Bio-Rad. RT-qPCR was performed using Roche SYBR Green master mix on a Roche LightCycler 480 Real-Time PCR System. qPCR primer sequences are listed in Supplementary Table S1.

### 2.5. RNA-seq and Analysis

RNA-seq libraries were prepared with the TruSeq RNA Library Prep Kit (Illumina, San Diego, CA, USA) and sequenced by the UCLA Technology Center for Genomics & Bioinformatics with an Illumina HiSeq 3000 system. Single-read datasets from mouse left ventricle RNA were mapped to the mm10 genome using Tophat v2.1.0 [39]. Single-read datasets from NRVM RNA were mapped to the rn6 genome using Tophat. Mapped reads were counted with FeatureCounts [40] and differential expression analysis was carried out with DESeq2 [41]. Gene ontology enrichment was examined using the R package clusterProfiler [42]. Plots were made using the R packages EnhancedVolcano and ComplexHeatmap [43,44,45].

### 2.6. Mice

All mice were maintained in the C57BL/6 background according to the Guide for the Care and Use of Laboratory Animals published by the US National Institute of Health. The mouse strain used for this research project, KOMP ES cell line Rtf1^tm1a(KOMP)Wtsi^, RRID:MMRRC_061682-UCD, was obtained from the Mutant Mouse Resource and Research Center (MMRRC) at the University of California at Davis, an NIH-funded strain repository, and was donated to the MMRRC by The KOMP Repository, University of California, Davis, originating from Pieter de Jong, Kent Lloyd, William Skarnes, Allan Bradley, Wellcome Sanger Institute. This line was crossed with a Flipase expressing strain (Jackson Laboratory, Bar Harbor, ME, USA) to create an Rtf1 conditional knockout allele where exon 3 of Rtf1 is flanked by two loxP sites (Rtf1^flox^). These mice were crossed into an Myh6:MerCreMer background [46] to produce Myh6:MerCreMer+;Rtf1^flox/flox^ animals for myocardium-specific knockout experiments. Knockout of Rtf1 was achieved by ad libitum feeding with TAM diet (TD.130859, Envigo, Indianapolis, IN, USA).

### 2.7. Histology Staining

Mouse hearts were fixed with 10% neutral buffered formalin and embedded in paraffin, sectioned along the short axis at 5 µm, and stained with hematoxylin and eosin by the UCLA Translational Pathology Core Laboratory. For AFOG staining, 5 µm paraffin sections were prepared by the UCLA Translational Pathology Core Laboratory, deparaffinized, incubated in Bouin’s Fixative for 2.5 h at 56 °C, incubated in 1% phosphomolybdic acid for 15 min, and then stained with AFOG solution (3 g Acid Fuchsin, 2 g Orange G, 1 g Anilin Blue dissolved in 200 mL acidified distilled water pH 1.09) overnight at room temperature.

### 2.8. Western Blotting

Mouse left ventricle tissue was lysed in RIPA lysis buffer (ThermoFisher #89901) supplemented with 1× Protease and Phosphatase Inhibitors (ThermoFisher #78440). Proteins were subjected to Polyacrylamide Gel Electrophoresis and transferred to a nitrocellulose membrane. Primary antibodies against Rtf1 (1:5000, Bethyl Labs #A300-179A, Montgomery, TX, USA) and H3 (1:5000, Abcam #ab1791, Cambridge, UK) were used along with HRP-conjugated secondary antibody (1:15,000, Zymax goat anti-rabbit HRP, ThermoFisher). ImageJ was used to perform densitometry comparing the level of Rtf1 protein between samples using histone H3 as the control.

### 2.9. Echocardiography

Mice were anesthetized with isoflurane and subjected to transthoracic echocardiography with a VisualSonics VEVO 2100 system once every 3–4 days. For each time point, data were acquired from 3–8 mice per condition. Left ventricular end-systolic and diastolic diameters were measured from M-mode echocardiograms of the left ventricle short axis. Ejection fraction and fractional shortening were calculated using the VEVO Lab Cardiac Analysis software package (VisualSonics, Toronto, ON, Canada).

### 2.10. Immunostaining of Mouse Heart Sections

Short axis cryosections of fresh unfixed mouse hearts were prepared by the UCLA Translational Pathology Core Laboratory. Following thawing and drying of sections, they were fixed with 4% PFA in PBS for 1 min. Sections were then blocked in 10% Goat serum in PBS for 1 h at room temperature, incubated with primary antibodies for 1 h at room temperature, and incubated with secondary antibodies for 30 min at room temperature. Antibodies used included anti-α-actinin (1:1000, clone EA-53, #A7732, Sigma-Aldrich), anti-connexin 43 (1:100, Cell Signaling #3512, Danvers, MA, USA), anti-N-cadherin (1:100, Developmental Studies Hybridoma Bank #MNCD2, Iowa City, IA, USA), anti-mouse IgG AlexaFluor 555 (1:1000, ThermoFisher #A31570), anti-rat IgG AlexaFluor 488 (1:1000, ThermoFisher #A21208), and anti-rabbit IgG AlexaFluor 555 (1:1000, ThermoFisher #A21428).

### 2.11. Statistics

Data in bar and line graphs are displayed as the mean ± the standard deviation. Statistical analysis for qPCR, Western, and echocardiography data was performed using Student’s *t*-test in Microsoft Excel, with a *p*-value of less than 0.05 considered to be significant. Statistics for differential gene expression were calculated via DESeq2, with an adjusted *p*-value of less than 0.01 considered to be significant.

## 3. Results

### 3.1. Rtf1 Regulates Neonatal Cardiomyocyte Morphology

**Figure 1:**
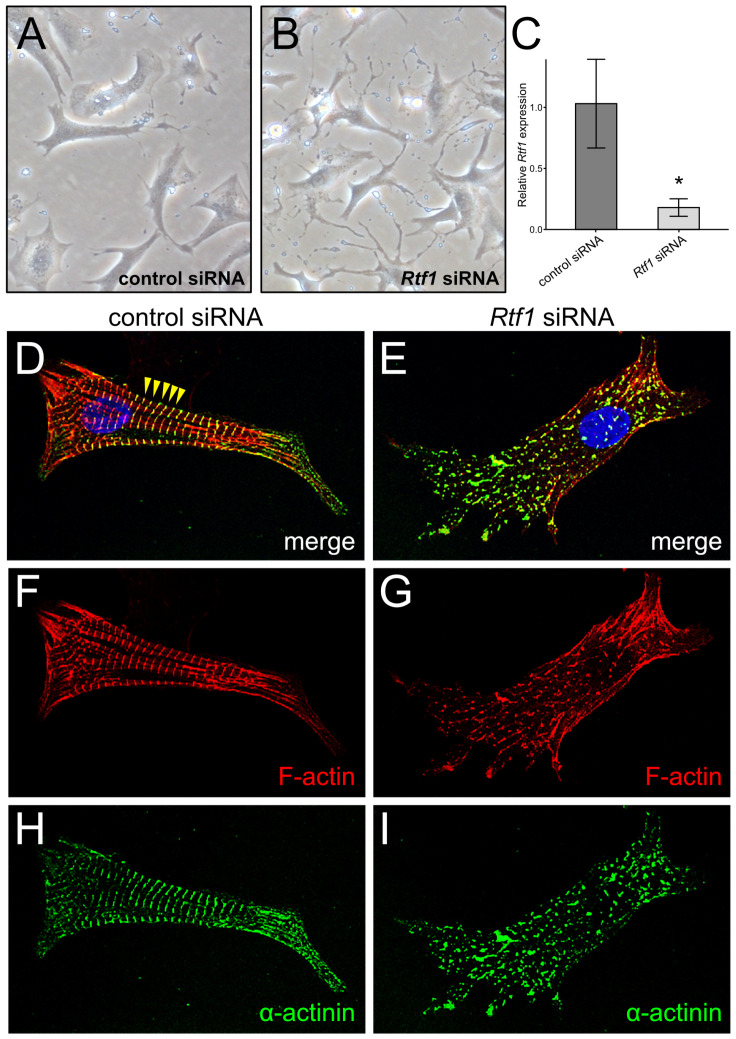
Rtf1 knockdown in NRVMs disrupts cell morphology. (**A**,**B**) Phase contrast microscopy of NRVMs treated with control and Rtf1 siRNAs. (**A**) Control siRNA-treated NRVMs have an elongated rod-like shape. (**B**) Rtf1 siRNA-treated NRVMs display filopodia-like protrusions. (**C**) Quantitative PCR analysis of Rtf1 expression in siRNA-treated NRVMs. * *p*-value < 0.05. (**D**–**I**) Fluorescent confocal microscopy of control and Rtf1 siRNA-treated NRVMs immunostained for α-actinin (green) and stained with phalloidin (red) and DAPI (blue). Yellow arrows point to representative Z-line sarcomere structures that are present in control siRNA-treated NRVMs (**D**) but not in Rtf1 siRNA-treated NRVMs (**E**).

To assess whether Rtf1’s transcription regulatory activity is needed for maintaining cardiomyocyte identity after it has been firmly established, we depleted Rtf1 transcripts in neonatal rat ventricular myocytes (NRVMs) using an siRNA knockdown approach. Rtf1 siRNA knockdown was highly effective, resulting in an 83% reduction of Rtf1 transcripts (Figure 1C). Interestingly, while no significant differences in cell survival were noted between NRVMs treated with Rtf1 targeting and control non-targeting siRNAs, Rtf1 knockdown induced a drastic change in NRVM morphology. Rtf1 siRNA-treated NRVMs have excessive cellular protrusions (Figure 1A,B), indicating alteration in cytoskeletal structure. Indeed, while phalloidin staining in control NRVMs was concentrated in actin fibers within sarcomeres and the termini of myofibrils (Figure 1D,F), Rtf1 siRNA-treated NRVMs lost these structures and displayed a disorganized actin cytoskeleton (Figure 1E,G). Furthermore, immunostaining showed that instead of localizing specifically to Z-lines, α-actinin staining in Rtf1 siRNA-treated NRVMs was punctate, reflecting a breakdown of sarcomere structure (Figure 1D,E,H,I). Together these data demonstrate that Rtf1 activity is essential for the structural maintenance of differentiated cardiomyocytes.

### 3.2. Inducible Knockout of Rtf1 in the Adult Mouse Myocardium

**Figure 2:**
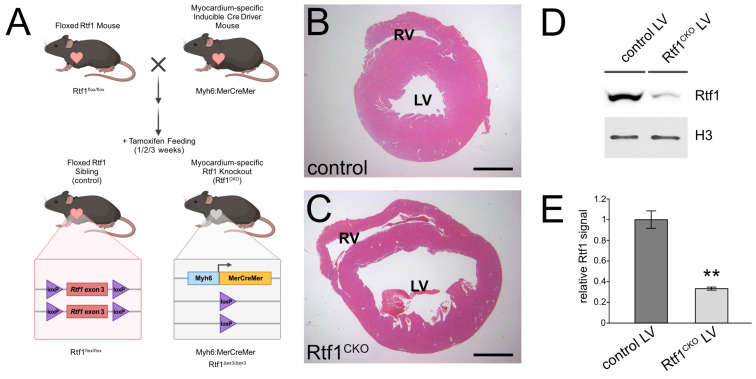
Generation of a cardiomyocyte-specific inducible Rtf1 knockout mouse. (**A**) Diagram of generation of cardiomyocyte-specific inducible Rtf1 knockout mice. A cardiomyocyte-specific Myh6:MerCreMer insertion allele was bred into an Rtf1 exon 3 floxed background. Homozygous Rtf1 floxed mice with or without Myh6:MerCreMer were fed with tamoxifen chow, inducing recombination and deletion of Rtf1 exon 3 in MerCreMer expressing hearts. (**B**,**C**) Hematoxylin and eosin stained paraffin sections of the short axis of control (**B**) and Rtf1^CKO^ (**C**) hearts after three weeks of tamoxifen feeding. Left (LV) and right (RV) ventricle chambers are labeled. (**D**) Western blot of Rtf1 protein and histone H3 protein (loading control) from left ventricle protein lysates isolated from control and Rtf1^CKO^ hearts after three weeks of tamoxifen feeding. (**E**) Quantification of Rtf1 protein levels based on Western blotting of left ventricle protein lysates. ** *p*-value < 0.01.

We next examined whether and how Rtf1 loss of function would impact the mature myocardium of the adult heart. To test this, we generated a mouse line carrying a floxed allele of Rtf1 and crossed it with a transgenic line expressing tamoxifen-inducible Cre under the control of the endogenous Myh6 (αMHC) promoter [46]. We fed MerCreMer-positive Rtf1 flox homozygous mice and their MerCreMer-negative Rtf1 flox homozygous siblings with tamoxifen chow for 1–3 weeks to generate Rtf1 conditional knockouts (Rtf1^CKO^) and control animals, respectively (Figure 2A). Western blotting of left ventricle protein demonstrated that Rtf1^CKO^ animals exhibited strongly reduced Rtf1 protein levels compared to controls (67% reduction, Figure 2D,E). Rtf1^CKO^ mice did not survive longer than 3 weeks after the initiation of Rtf1 knockout (data not shown), and their hearts exhibited pronounced dilation with thinning of the ventricular walls (Figure 2B,C).

### 3.3. Rtf1-Deficient LV Exhibits Structural Deterioration and Disruption of Intercalated Discs

**Figure 3:**
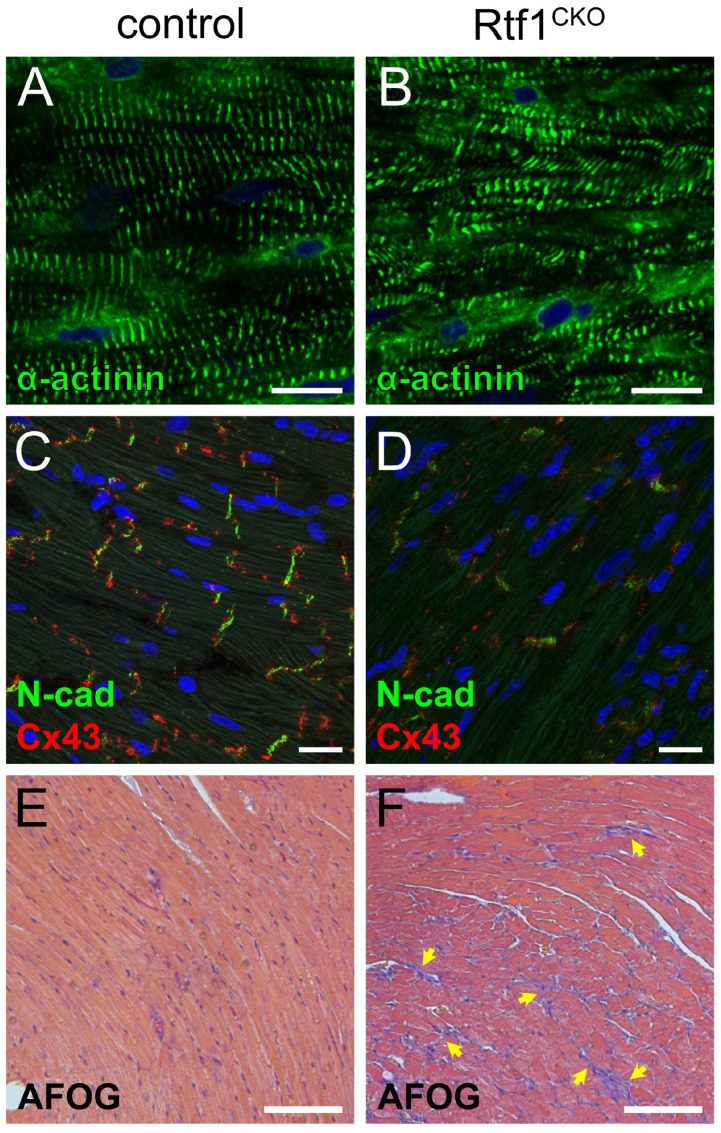
Rtf1-deficient LV exhibits structural deterioration and disruption of intercalated discs. (**A**,**B**) Immunostaining for α-actinin (green) on cryosections of left ventricle tissue. Nuclei are labeled with DAPI (blue). Regular parallel lines of α-actinin staining are visible in control LVs (**A**), while disorganized α-actinin staining is present in Rtf1^CKO^ LVs (**B**). (**C**,**D**) Immunostaining for N-cadherin (N-cad; green) and Connexin-43 (Cx43; red) proteins on cryosections of left ventricle tissue. Nuclei are labeled with DAPI (blue). Cell–cell junctions are visible in control LVs (**C**), marked by N-cad and Cx43 staining, whereas Rtf1^CKO^ LVs have very reduced staining for N-cad and Cx43 (**D**). (**E**,**F**) AFOG staining of paraffin sectioned left ventricle tissue. Yellow arrows point to large areas of collagen protein deposition (blue/purple). Control LV myocardium consists primarily of cardiomyocytes (orange) (**E**). Rtf1^CKO^ myocardium is fibrotic with large inclusions of collagen protein (**F**).

To gain a deeper understanding of the cellular and tissue-level changes resulting from loss of Rtf1 activity in the adult myocardium, we examined sections of Rtf1^CKO^ and control hearts after 3 weeks of tamoxifen feeding via immunostaining and histological staining. Both Rtf1^CKO^ and control LVs displayed parallel lines of α-actinin staining, indicating that Z-lines of sarcomeres were intact in Rtf1 knockout hearts. However, myofibrils were more disorganized in Rtf1^CKO^ LVs compared to controls (Figure 3A,B). Strikingly, we found that junctional proteins of the intercalated disc were strongly reduced in the Rtf1^CKO^ heart. Control LVs displayed strong adjacent, but largely non-overlapping, signals for N-cadherin (adherens junctions/area composita) and Connexin-43 (gap junctions) between myocytes (Figure 3C). Rtf1^CKO^ LVs, on the other hand, had patchy, weak N-cadherin and Connexin-43 signals (Figure 3D), suggesting altered cell–cell junctions.

We also used Acid Fuchsin Orange G (AFOG) staining to examine the myocardium and extracellular matrix of Rtf1^CKO^ LVs. AFOG stains cardiomyocytes orange and extracellular matrix blue (collagen) and red (fibrin). Control myocardium was composed of primarily orange-stained cardiomyocytes with occasional small vessels surrounded by fibrin signal (Figure 3E). Rtf1^CKO^ LV myocardium exhibited a dramatic increase in cardiomyocyte-free regions filled primarily with collagen, suggesting the presence of fibrosis (Figure 3F). Together, these data demonstrate the Rtf1-deficiency-induced heart failure is characterized by disrupted myofibril organization, defective intercalated disc structure, and widespread fibrosis.

### 3.4. Myocardial Knockout of Rtf1 Causes Progressive Dilated Cardiomyopathy and Severe LV Systolic Dysfunction

**Figure 4:**
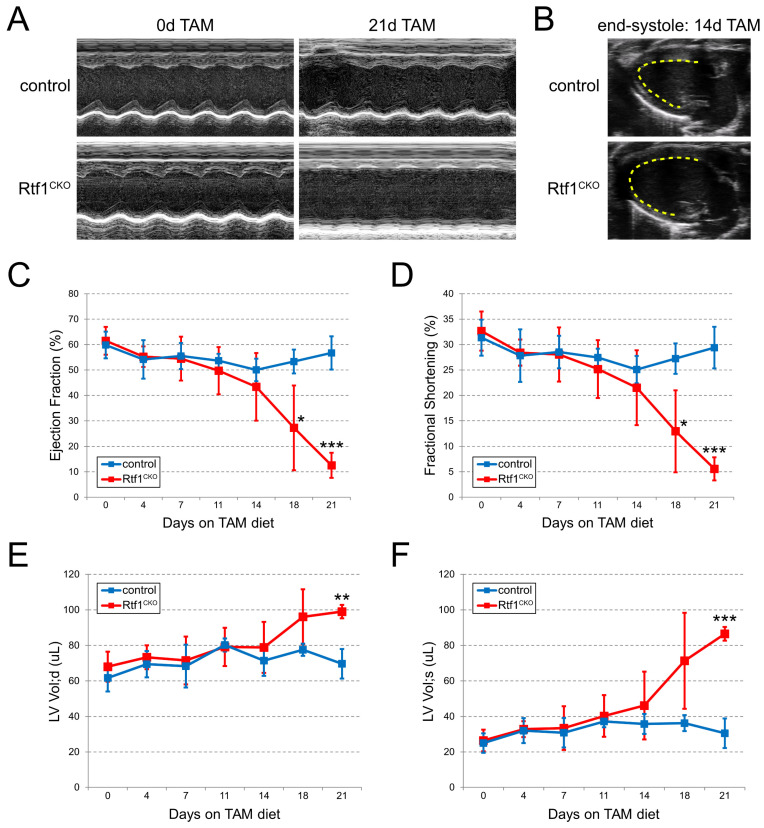
Myocardial knockout of Rtf1 causes dilated cardiomyopathy and LV systolic dysfunction. (**A**) Short-axis M-mode echocardiograms of control and Rtf1^CKO^ ventricles prior to tamoxifen feeding and after 21 days of tamoxifen feeding. (**B**) Long-axis B-mode echocardiograms of control and Rtf1^CKO^ hearts at the level of the left ventricle after 14 days of tamoxifen feeding. The inner wall of the LV is traced with a yellow dashed line. (**C**) Graph of ejection fraction values of control and Rtf1^CKO^ hearts from 0 to 21 days of tamoxifen feeding. (**D**) Graph of fractional shortening values of control and Rtf1^CKO^ hearts from 0 to 21 days of tamoxifen feeding. (**E**) Graph of LV volume at diastole values of control and Rtf1^CKO^ hearts from 0 to 21 days of tamoxifen feeding. (**F**) Graph of LV volume at systole values of control and Rtf1^CKO^ hearts from 0 to 21 days of tamoxifen feeding. Error bars in (**C**–**F**) represent standard deviations from the mean. *, **, *** denote *p*-values < 0.05, 0.01, and 0.001, respectively.

Rtf1-deficient adult hearts become dilated with fibrosis and defective junctions prior to complete failure of cardiac function. To better understand the progression of this heart failure phenotype, we performed echocardiography on Rtf1^CKO^ and control mice. Prior to feeding with tamoxifen and during the first week of tamoxifen feeding, both Rtf1^CKO^ and control animals exhibited normal left ventricular contractile parameters including ejection fraction (EF), fractional shortening (FS), diastolic volume (LV Vol; d), and systolic volume (LV Vol; s) (Figure 4A,C–F). However, after two weeks of tamoxifen treatment, Rtf1^CKO^ hearts displayed a trend of reduced left ventricular function (Figure 4C–F) and visible dilation reflecting compromised contractility (Figure 4B). After treatment with tamoxifen for 3 weeks, all Rtf1^CKO^ hearts were dilated, particularly at systole, and displayed dramatic and significantly decreased ventricular contractility (6% FS in Rtf1^CKO^ vs. 29% FS in controls, *p* = 0.00029; Figure 4A,C–F). Taken together, these data suggest that loss of Rtf1 in the adult myocardium causes progressive left ventricular systolic dysfunction culminating in dilated cardiomyopathy and heart failure.

### 3.5. Rtf1 Knockout LV Exhibits Progressive Transcriptional Dysregulation

**Figure 5:**
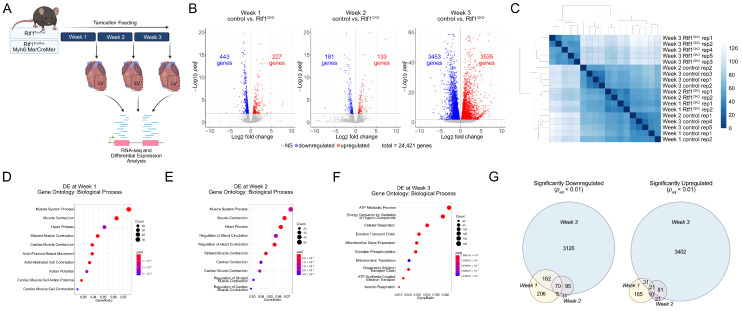
Rtf1 knockout in the adult heart causes progressive transcriptional dysregulation. (**A**) Schematic overview of Rtf1 adult myocardium knockout RNA-seq experiment. (**B**) Volcano plots visualizing log2 (fold changes) vs. −log10 (adjusted *p*-values) for comparisons of control and Rtf1^CKO^ LV samples at weeks 1, 2, and 3 following initiation of tamoxifen feeding. Dots representing genes with significantly altered expression (adjusted *p*-value of 0.01 or less) are in color (blue: downregulated; red: upregulated). (**C**) Sample-to-sample Euclidean distance heatmap displaying similarity between replicates of each condition. Distances were calculated from the variance stabilized transformation of the data. (**D**–**F**) Plots of enriched Biological Process gene ontology terms for the sets of genes that were differentially expressed at Week 1 (**D**), 2 (**E**), or 3 (**F**). Dot sizes indicate the number of genes associated with a given ontology term. Dot color indicates the significance level (adjusted *p*-value). (**G**) Venn diagrams showing common and differing genes that were differentially expressed at each time point of RNA-seq sample collection.

We sought to understand the gene expression changes resulting from loss of Rtf1 activity in the adult myocardium, including both early/primary and late/secondary transcriptional responses to Rtf1 deficiency that underly the dramatic heart failure phenotype we observed. To examine this, we isolated RNA from left ventricle tissue of Rtf1^CKO^ and control mice at 1, 2, and 3 weeks after initiating tamoxifen feeding and performed RNA-seq and differential expression analysis (Figure 5A). Hierarchical clustering showed that RNA samples from Rtf1^CKO^ LVs were clearly grouped and distinct from control LVs regardless of the time point analyzed (Figure 5C). Correspondingly, Rtf1^CKO^ LVs exhibited significant changes in gene expression at all three time points, including similar amounts of upregulated and downregulated genes (Tables S2–S4). We focused on those differentially expressed genes with an adjusted *p*-value of less than 0.01 (1% false discovery rate) for further analysis (Figure 5B).

To explore the biological processes and pathways that were altered in Rtf1^CKO^ LVs, we performed gene set enrichment tests of the significantly differentially expressed genes at each RNA isolation time point. Genes involved in striated/cardiac muscle contraction (Biological Process gene ontologies) were highly enriched in week 1 and 2 Rtf1^CKO^ LVs (Figure 5D,E), suggesting that early gene expression changes upon loss of Rtf1 activity contributed to the emerging altered cardiac contractility we observed via echocardiography. Interestingly, genes involved in mitochondrial biology and metabolism (Biological Process gene ontologies) were highly enriched at week 3 (Figure 5F), suggesting that the heart failure phenotype in Rtf1^CKO^ animals is partly the result of disrupted mitochondrial function.

### 3.6. Failing Rtf1 Knockout Hearts Exhibit Gene Expression Changes Similar to Those Observed in Dilated Cardiomyopathy

**Figure 6:**
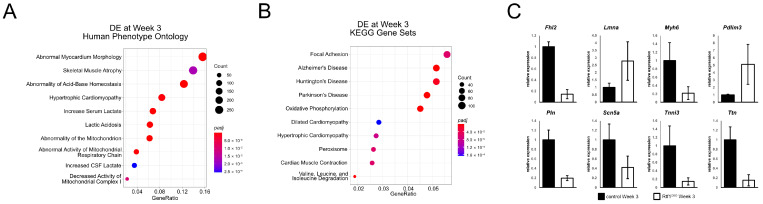
Failing Rtf1 knockout hearts exhibit gene expression changes similar to those observed in dilated cardiomyopathy. (**A**) Plot of enriched Human Phenotype ontology terms among the genes that were differentially expressed between control and Rtf1^CKO^ LVs after 21 days of tamoxifen feeding. (**B**) Plot of enriched KEGG gene sets among the genes that were differentially expressed between control and Rtf1^CKO^ LVs after 21 days of tamoxifen feeding. Dot sizes in (**A**,**B**) indicate the number of genes associated with a given ontology term. Dot color in (**A**,**B**) indicates the significance level (adjusted *p*-value). (**C**) Quantitative PCR analysis of selected human dilated cardiomyopathy associated genes on LV samples from control and Rtf1^CKO^ mice fed with tamoxifen chow for three weeks.

To further examine the gene expression changes associated with Rtf1 knockout-induced heart failure, we tested the set of genes that was differentially expressed three weeks following the induction of Rtf1 knockout for enrichment of Human Phenotype and KEGG gene set ontologies. Consistent with the structural deterioration we observed in Rtf1^CKO^ hearts, we found a highly significant enrichment of genes associated with abnormal myocardium morphology (Figure 6A). Intriguingly, the KEGG dilated cardiomyopathy (DCM)-associated gene set was also enriched among week 3 differentially expressed genes (Figure 6B). We selected several genes that have been linked to DCM in human patients [47,48,49] and performed qPCR, confirming that they are indeed significantly altered in Rtf1^CKO^ hearts (Figure 6C). Together, these results demonstrate that loss of Rtf1 activity in the adult mouse myocardium not only induces functional changes like those observed in human dilated cardiomyopathy, but also gene expression changes as well.

### 3.7. Rtf1 Transcriptionally Regulates a Core Set of Critical Cardiac Genes

**Figure 7:**
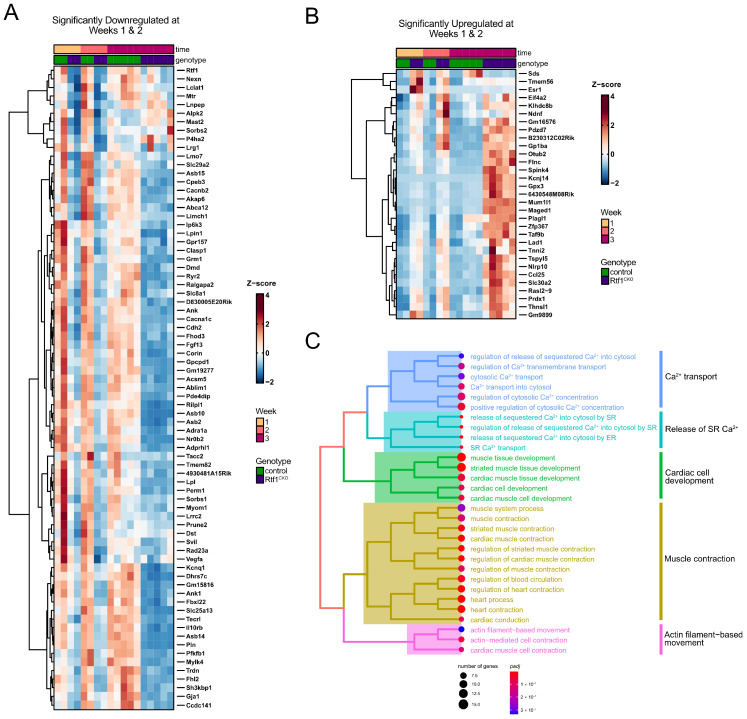
Rtf1 regulates a core set of cardiac structural and functional genes in adult cardiomyocytes. (**A**,**B**) Heatmaps of scaled expression levels of genes that are significantly downregulated (**A**) or upregulated (**B**) in Rtf1^CKO^ LVs at both the one- and two-week time points of RNA-seq sample collection following initiation of tamoxifen feeding. (**C**) Hierarchical clustering of significantly enriched Biological Process gene ontology terms for the set of genes that is significantly downregulated in Rtf1^CKO^ LVs at both the one- and two-week timepoints of RNA-seq sample collection following initiation of tamoxifen feeding.

Given the similar biological processes disrupted at one and two weeks of myocardial Rtf1 knockout, which occur prior to the functional deterioration of the heart after three weeks of knockout, we hypothesized that the associated genes represent a core set of Rtf1-dependent transcriptional regulatory targets. Indeed, we found substantial overlap between the differentially expressed genes at one and two weeks following the initiation of Rtf1 knockout (Figure 5G). A total of 75 genes were significantly downregulated at both weeks 1 and 2, and 70 of those genes continued to be downregulated at week 3 (Figure 5G and Figure 7A). A total of 31 genes were significantly upregulated at both of the first two time points, of which 21 were also upregulated at week 3 (Figure 5G and Figure 7B). As expected, we observed that the Rtf1 gene was significantly downregulated at 1, 2, and 3 weeks after beginning tamoxifen treatment (Figure 7A). Additionally, we found that numerous channels (Ryr2, Kcnq1, Slc8a1, Cacnb2, Kcnj14), intercalated disc proteins (Gja1, Cdh2), and sarcomere components (Myom1, Tnni2) were also present in the core set of Rtf1-dependent genes (Figure 7A,B), indicating that targets of Rtf1 activity include genes directly involved in both cardiac structure and function. Importantly, early downregulation of the Gja1 and Cdh2 genes also indicates that the loss of gap junction and adherens junction/area composita signal for Connexin-43 and N-cadherin proteins in Rtf1 knockout myocardium is caused directly by changes in transcript abundance upon Rtf1 ablation rather than secondary changes leading to protein degradation. We further analyzed the biological functions of Rtf1’s regulatory targets via hierarchical clustering and found that the biological processes most significantly dysregulated upon loss of Rtf1 include calcium ion transport, release of calcium stores from the sarcoplasmic reticulum, muscle contraction, actin filament-based movement, and cardiac muscle developmental pathways (Figure 7C). Together, these data demonstrate that Rtf1 transcriptionally regulates a core set of genes essential for maintaining the structure and function of the adult heart.

### 3.8. Rtf1 Regulates Genes Essential for Neonatal Cardiomyocyte Biology

**Figure 8:**
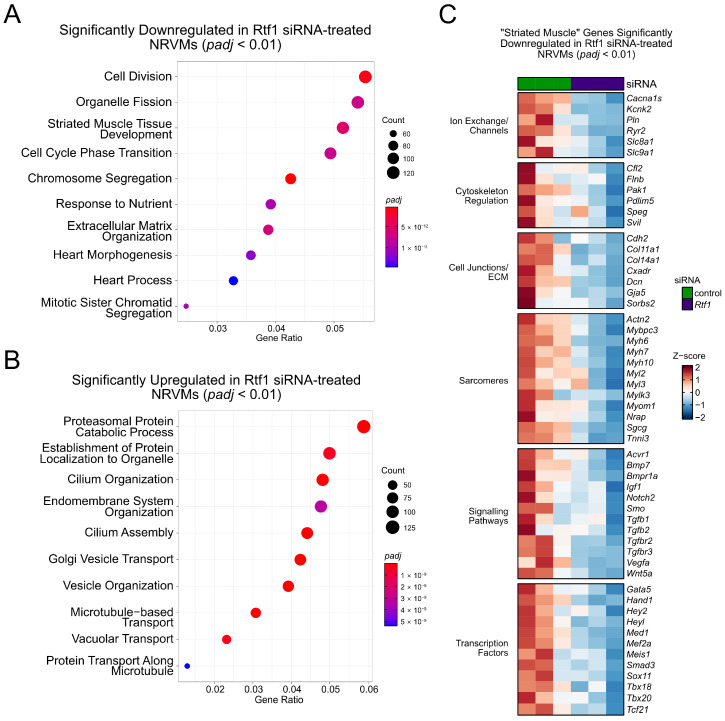
Rtf1 regulates genes essential for neonatal cardiomyocyte biology. (**A**,**B**) Plots of enriched Biological Process gene ontology terms for the sets of genes that were significantly downregulated (**A**) and upregulated (**B**) in NRVMs treated with Rtf1 siRNA. Dot sizes in A and B indicate the number of genes associated with a given ontology term. Dot color in A and B indicates the significance level (adjusted *p*-value). (**C**) Heatmap of scaled expression levels of selected genes that are significantly downregulated in Rtf1 siRNA-treated NRVMs. Heatmap is divided into sections based on the biological functions of gene products.

We also performed RNA-seq to examine the transcriptomic changes underlying the cellular defects we observed in Rtf1-depleted NRVMs. Our differential expression analysis showed that genes involved in catabolism and the microtubule cytoskeleton are highly enriched among those genes upregulated in Rtf1 siRNA-treated cells (Figure 8B), whereas genes involved in cell division, extracellular matrix organization, and striated muscle/heart development are highly enriched among significantly downregulated genes (Figure 8A, Table S5). Intriguingly, and consistent with the early gene expression changes we observed in Rtf1^CKO^ LVs, we found that genes critically involved in cardiac biology including ion exchangers/channels, cytoskeleton regulators, transcription factors, and components of cell junctions/ECM, sarcomeres, and signaling pathways were among the “striated muscle”-related downregulated genes in Rtf1 knockdown cells (Figure 8C). Overall, these genetic changes show that Rtf1 activity is continuously required for post-embryonic maintenance of the cardiac gene program in neonatal and adult cardiomyocytes.

## 4. Discussion

Each step in the transcription cycle, from initiation to termination, is subject to regulatory controls that impact the final expression level of a given gene. Rtf1, a member of the evolutionarily conserved PAF1C, regulates transcription at several steps and has been shown to influence a range of developmentally relevant genetic pathways. Our previous finding that Rtf1 is required for cardiac progenitor formation suggested that Rtf1 may be a key regulator of the cardiac gene program [19]. In this current study, we tested whether Rtf1 is required in neonatal and adult cardiomyocytes. Interestingly, we found that both neonatal and adult Rtf1-ablated cardiomyocytes display downregulation of critical cardiac structural and functional genes. Consequently, the cellular morphology of neonatal cardiomyocytes and the structure and function of the adult heart are severely disrupted upon loss of Rtf1.

### 4.1. Genetic Regulation of Dilated Cardiomyopathy

Rtf1 knockout hearts are dilated and only very weakly contract, resembling human dilated cardiomyopathy. Rtf1-deficient hearts also exhibit severe defects in intercalated disc structure based on the diminished and diffuse localization of N-cadherin and Connexin-43 proteins (Figure 3C,D). Interestingly, intercalated disc abnormalities and decreased N-cadherin protein have been associated with dilated cardiomyopathy in human patients [50,51], suggesting that Rtf1 knockout hearts also have cellular-level similarities to dilated cardiomyopathy. Consistent with these phenotypic parallels, we also identified a significant enrichment in genes associated with dilated cardiomyopathy among those genes that were differentially expressed after 3 weeks of Rtf1 loss of function (Figure 6B,C). While changes in Rtf1 expression or variants in Rtf1 have not been clinically associated with DCM or other types of heart failure, our data suggest that loss of Rtf1 may be able to model some types of DCM, in particular those caused by changes in intercalated discs, ion exchange, or sarcomere structure.

### 4.2. Maturation of Differentiated Cardiomyocytes

Neonatal and adult cardiomyocytes differ in several respects. Maturation of myofibril structure, electrophysiological properties, and metabolism occur in adult cardiomyocytes, and in contrast to neonatal cardiomyocytes, adult cardiomyocytes lack proliferative capacity and exhibit hypertrophic growth [52]. These structural and functional changes are directed by the interplay of biophysical/environmental cues and transcriptional regulation [52,53,54]. We observed similar gene expression changes in Rtf1 knockdown neonatal cardiomyocytes and Rtf1 knockout adult left ventricles, including decreased expression of channels, sarcomere contractile components, and junctional components. NRVMs lacking Rtf1 activity also displayed a significant decrease in the expression of genes involved in cell division (Figure 8A), suggesting that Rtf1 regulates the proliferative capacity of neonatal cardiomyocytes. On the other hand, and consistent with the non-proliferative nature of adult mouse cardiomyocytes, Rtf1 knockout adult hearts did not exhibit significant enrichment for cell division genes among significantly downregulated genes at any time point. Interestingly, we did observe an increase in the expression of cell division regulators and fibroblast migration genes at 3 weeks following Rtf1 ablation (data not shown). While further studies would be required to identify the cell types upregulating cell division genes in the Rtf1 knockout left ventricle, we hypothesize these gene expression changes may be pathogenic and reflect fibrosis in the myocardium during heart failure.

Intriguingly, we also observed a difference between the integrity of neonatal and adult sarcomeres upon loss of Rtf1 activity. While the adult Rtf1^CKO^ myocardium does exhibit myofibril disorganization (Figure 3B), Z-lines are still present, suggesting the existence of intact sarcomeres. In contrast, Rtf1 siRNA-treated NRVMs displayed a dramatic loss of α-actinin-positive Z-lines (Figure 1I). Cardiomyocytes within the adult heart possess cell–cell and cell–matrix attachments and express more mature forms of sarcomere components than neonatal cardiomyocytes [52,55]. Given these differences and the dramatic cytoskeletal changes and cellular protrusions we consistently observed in Rtf1 siRNA-treated NRVMs, we favor the hypothesis that Rtf1 deficiency-induced gene expression changes may be more detrimental to sarcomere homeostasis in isolated cultured neonatal cardiomyocytes than in mature adult cardiomyocytes. Alternatively, given the proliferative nature of neonatal cardiomyocytes [56], the absence of Z-lines in Rtf1 siRNA-treated NRVMs may reflect a defect in reestablishing sarcomere structures following mitosis.

### 4.3. Transcriptional Regulation of Cardiac Gene Expression

Our finding that genes relevant to cardiac biology are highly enriched among those genes downregulated upon Rtf1 ablation suggests that Rtf1 positively regulates this set of genes. As an elongation factor, Rtf1 may directly promote the expression of cardiac genes by supporting RNA Pol II processivity. Indeed, diminished RNA Pol II processivity caused by loss of the PAF1C component Paf1 was found to decrease expression of a subset of genes, and strongly impact the transcription of long genes in particular [57,58]. Rtf1’s key role in H2Bub1 deposition and the deposition of H3K79me3 and H3K4me3 marks, which are influenced by the presence of H2Bub1, may also be required for supporting the expression of its target genes. Intriguingly, human patients carrying mutations and deletions of UBE2A, a human homolog of the yeast E2 ubiquitin conjugating enzyme Rad6, exhibit severe intellectual disability and occasional cardiac malformations [59], suggesting that Rtf1-dependent H2B monoubiquitination may regulate the cardiac gene program.

Rtf1 may also promote the expression of cardiac genes by influencing the promoter-proximal pausing of RNA Pol II. During neural crest development, Rtf1 opposes the activity of Cdk9/P-TEFb, a key factor that promotes the transition of RNA Pol II from a paused state to processive elongation, suggesting that Rtf1 promotes promoter-proximal pausing at genes regulating development of the neural crest lineage [18]. Similarly, Rtf1 loss of function suppresses the erythroid differentiation defects caused by an absence of TIF1γ, a transcription factor required for recruiting positive elongation factors to erythroid genes. This suggests that in the context of erythroid gene expression, Rtf1 acts as a negative regulator of transcription elongation possibly by promoting pausing [21]. Intriguingly, promoter-proximal pausing is capable of both facilitating and restraining gene expression [3,60,61,62,63,64,65,66,67,68], suggesting that Rtf1 activity may promote the expression of cardiac genes or repress the expression of a negative regulator of cardiac gene networks. Given Rtf1’s ability to influence multiple steps in the transcription cycle, the downregulation of genes essential for cardiac structure and function that occurs upon loss of Rtf1 activity likely results from a combination of transcriptional regulatory defects.

Our study provides the first evidence that Rtf1-dependent transcriptional regulatory activity is continuously required in the myocardial lineage, from embryonic stages to adulthood. Loss of Rtf1 function impacts the cellular structure and gene expression of neonatal cardiomyocytes and the structure and function of the adult heart. Future investigation of the specific transcription regulatory mechanisms influenced by Rtf1 is likely to provide basic information about how cardiac genes are regulated and may lead to therapeutic advances for treating heart disease.

## Data Availability

The RNA-seq data presented in this study are openly available in the NCBI SRA (BioProjects PRJNA957813 and PRJNA957539).

## Supplementary Materials

The following supporting information can be downloaded at: https://www.mdpi.com/article/10.3390/jcdd10050221/s1, Table S1: Primers used for quantitative real-time PCR, Table S2: DESeq2 differential gene expression analysis comparing control and Rtf1^CKO^ left ventricles after one week of tamoxifen feeding, Table S3: DESeq2 differential gene expression analysis comparing control and Rtf1^CKO^ left ventricles after two weeks of tamoxifen feeding, Table S4: DESeq2 differential gene expression analysis comparing control and Rtf1^CKO^ left ventricles after three weeks of tamoxifen feeding, Table S5: DESeq2 differential gene expression analysis comparing control and Rtf1 siRNA-treated NRVMs.
