# Supplementary material for: Rtf1 Transcriptionally Regulates Neonatal and Adult Cardiomyocyte Biology
Source: J Cardiovasc Dev Dis. 2023 May 20;10(5):221. doi: 10.3390/jcdd10050221 (PMC10219292; doi:10.3390/jcdd10050221)
Supplement: Supplementary file 1 [file jcdd-10-00221-s001.zip › Table S1.pdf]

Table S1. Primers used for quantitative real-time PCR

Gene	Forward Primer	Reverse Primer
**rPPIA**	GTCAACCCCACCGTGTTCTTC	ATCCTTTCTCCCCAGTGCTCAG
**mRtf1**	CAGTGATGATGAGTGGACATT	CTTTGTTGGCTTGCTTCTTC
**mFhl2**	ATGACTGAACGCTTTGACTGC	CGATGGGTGTTCCACACTCC
**mLmna**	GGATGCTGAGAACAGGCTACA	CTCTCGCTGCTTCCCGTTATC
**mMyh6**	GCCCAGTACCTCCGAAAGTC	GCCTTAACATACTCCTCCTTGTC
**mPdlim3**	TGGGGGCATAGACTTCAATCA	CTCCGTACCAAAGCCATCAATAG
**mPln**	CCTTCCTGGCATAATGGAAA	CATGTTGCAGGTCTGGAGTG
**mScn5a**	ATGGCAAACTTCCTGTTACCTC	CCACGGGCTTGTTTTTCAGC
**mTnni3**	TCTGCCAACTACCGAGCCTAT	CTCTTCTGCCTCTCGTTCCAT
**mTtn**	CTACGTGGTAGAAAAGCGAGAAA	ACACCGTACTTGTTGACAGCC
**mEef1e1**	TCCAGTAAAGAAGACACCCAGA	GACAAAACCAGCGAGACACA
